# An international unified approach to reporting and grading invasive breast cancer. An overview of the International Collaboration on Cancer Reporting (ICCR) initiative

**DOI:** 10.1111/his.14802

**Published:** 2022-12-08

**Authors:** Ian O Ellis, Emad A Rakha, Gary M Tse, Puay Hoon Tan

**Affiliations:** ^1^ Translational Medical Sciences, School of Medicine, The University of Nottingham, Department of Histopathology, City Hospital Campus Nottingham University Hospitals Nottingham UK; ^2^ Department of Anatomical and Cellular Pathology, Prince of Wales Hospital The Chinese University of Hong Kong Shatin Northwest Territories Hong Kong SAR; ^3^ Department of Anatomical Pathology Singapore General Hospital Singapore

**Keywords:** Breast cancer, reporting, grading, guidelines, approaches

## Abstract

Standardised reporting of breast cancer key pathology data has become the norm in some parts of the world, but are based on national or regional guidelines that differ in certain aspects, resulting in divergent reporting practices and a lack of comparability of data internationally. The International Collaboration on Cancer Reporting (ICCR), a global alliance of major (inter‐)national pathology and cancer organizations, have recently produced a new international dataset for the pathology reporting of breast cancer, including resection specimens with invasive cancer and ductal carcinoma *in situ* (DCIS) of the breast. This initiative aims at providing an international unified approach to reporting cancer. The guidance was prepared by an international expert panel consisting of experienced breast pathologists, a surgeon, and an oncologist. The dataset includes core (essential) and noncore (optional) data items based on a critical review and discussion of current evidence. Commentary is provided for each data item to explain the rationale for selection, its clinical relevance, and to highlight potential areas of disagreement or lack of evidence, in which case a consensus position was formulated. The process concludes with international public consultation, before ratification and publication on the free open access ICCR website, with a synoptic reporting guide. The key aim is to promote high‐quality, standardised pathology reporting that can be used worldwide. Histological grade, tumour size, and oestrogen receptor status are used in this article to illustrate this process and the detail provided to support its inclusion.

## Graphical abstract

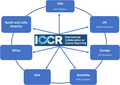

## Background and unmet needs

To reduce mortality from breast cancer worldwide requires all professional groups involved to perform to the highest standards. The quality of pathological services underpins breast cancer care and is of the utmost importance; it is the pathologist who invariably makes the definitive diagnoses of breast cancer but additional features of *in situ* and invasive carcinomas that have prognostic significance are also required to determine the most appropriate management for individual patients. Therefore, accurate pathology diagnoses and the provision of prognostically significant information are important, not only to ensure that patients are managed appropriately, but also for breast services to be effectively monitored and evaluated. For these fundamental reasons the management of patients with breast disease and breast cancer depends heavily on the quality of the pathology service.

Global standardization of pathology information forms the basis for best practice in patient care and is a prerequisite for epidemiological research and benchmarking in cancer management both nationally and internationally. The global standardization of classification of tumours is currently led by the International Agency for Research on Cancer (IARC), which is part the World Health Organization of the United Nations,^1^ while the staging is led by the Union for International Cancer Control (UICC),^2^ and the American Joint Committee on Cancer (AJCC)^3^ and these have gained wide international acceptance.

A standard set of data from each patient, using the same terminology and diagnostic criteria, is essential to improve the quality and completeness of a pathology cancer report.^4^ Over the last 30 years various national organizations including the Royal College of Pathologists in the United Kingdom (UK) (RCPath) (https://www.rcpath.org/),^5^ the United States (US) College of American Pathologists (CAP) (https://www.cap.org/),^6^ the Royal College of Pathologists of Australasia (RCPA) (https://www.rcpa.edu.au/),^7^ the European Society of Pathology (ESP) (https://www.esp‐pathology.org/) and other national organization and colleges such as the National Health Commission of the People's Republic of China have produced reporting guidelines. These documents, which have been updated on a regular basis, aim to give guidance and recommendations on all aspects of pathology examination of breast lesions and are relevant to both screen‐detected and symptomatic disease. These guidelines therefore aim to encourage the use of common terminology and definitions of breast disease and methods of classifying breast cancer in their specific regions, but inevitably differ subtly in their content and generally have expectations or assumptions of the resource available to their target pathologists.

These reporting datasets, which guide reporting of breast cancer, have been implemented for the following reasons.

1. Certain features of invasive carcinoma (e.g. tumour type, size, grade, lymphovascular invasion, and lymph node status) have been shown to be related to clinical outcome. Consequently, these features may be important in:

- deciding on the most appropriate treatment for a particular patient, including the extent of surgery and the use and choice of adjuvant therapy;
- monitoring breast screening programmes, the success of which is reflected by more favourable prognostic features of the cancers detected;
- monitoring changing patterns of disease, particularly by cancer registries.

1. Classification of ductal carcinoma *in situ* (DCIS) together with reporting of margins of excision and size has been shown to be related to the probability of recurrence after local excision and may influence the use of mastectomy or adjuvant radiotherapy.
2. Close correlation of radiological and histopathological features is essential to ensure that mammographically detected lesions have been sampled and accurately diagnosed.
3. Guidance and recommendation on standardization and quality of laboratory evaluation are key to reporting predictive factors, specifically hormone receptor and HER2 status.

These national and regional guidelines and datasets, to a degree, result in duplication of advice in many Western countries including Europe, and may have differences or appear contradictory. This availability and ease of access to information contrasts with other regions including Africa, and large parts of Asia and South America, where there are no established datasets or guidance. There has been a perceived need for international agreement on guidelines that are standardised, particularly with respect to dataset elements, naming conventions, value list units, and methods of measurement and explanatory text.

## The International Collaboration on Cancer Reporting (ICCR) initiative

The International Collaboration on Cancer Reporting (ICCR) (https://www.iccr‐cancer.org/) is a nonprofit organization incorporated in 2014 to develop internationally standardised and evidenced‐based checklists for the pathology reporting of cancer with the aim to advance international benchmarking in cancer management. Now with 12 sustaining members, the ICCR has representation on five continents.

The ICCR's goal is to develop datasets, in sync with the WHO Bluebooks publication cycle,^1^ for the most commonly diagnosed cancers worldwide, which account for ~90% of all reported cancers. While the datasets are intended to provide pathologists worldwide with evidence‐based checklists for the reporting of cancer, as stated above, it is likewise beneficial to have standardised datasets for cancer surveillance and registry purposes.

The use of standardised checklists enables pathologists to keep abreast of the latest information relevant to each cancer. While the Colleges of Pathology of the USA, UK, and Australasia and other organizations around the world have produced their own standardised cancer reporting datasets for national use,^5^, ^6^, ^7^ these organizations along with the Canadian Association of Pathologists‐Association Canadienne des Pathologists (CAP‐ACP) (https://www.cap‐acp.org/) in association with the Canadian Partnership Against Cancer (CPAC) (https://www.partnershipagainstcancer.ca/) and the European Society of Pathology, have realised the benefits of aligning pathology cancer dataset development internationally and together formed the ICCR.

The production of ICCR datasets can significantly reduce the development effort for those organizations developing their own cancer checklists such as CAP and RCPath and allows these countries to align and normalize their pathology cancer data. The protocols also provide a means of achieving an international standard of pathology reporting without significant investment amongst many countries, especially the low‐ and middle‐income ones, which lack the resources for their own cancer dataset development.

Several of the ICCR datasets have been translated into other languages, including Spanish, French, and Portuguese, thus facilitating usage in countries where English is not the reporting language. There are also plans to translate the datasets into other languages including German, Russian, and Chinese.

In time, as the ICCR datasets are adopted, more accurate, standardised data will be sent to the cancer registries, allowing better accuracy in comparisons of diagnostic, prognostic, and predictive cancer pathology information worldwide.

### What constitutes an ICCR dataset?

An ICCR dataset is an accessible publication of evidence‐based protocols for the pathology reporting of each organ‐specific cancer as structured data. They aim to improve clinical practice, facilitating ‘buy in’ from pathologists and supporting quality assurance.

The ICCR provides the capability for database development and potential electronic implementation of these protocols as discrete data, which can be applied across large populations, enabling aggregation and automated analysis of data in real time, supporting evaluation of epidemiological and other research quality indicators and public health management.

The benefits of a universal approach include:

- Utilization of the world's best expertise with high‐level domain knowledge creating a one‐world resource.
- Developing countries have access.
- Harmonization of terminology.
- Interoperability of health data internationally.
- Cost reduction.
- Burden on expert resources shared.
- Potential for simplified IT implementation in the laboratory.

Success of the ICCR initiatives will be seen as:

- Unified global language for reporting all cancers (50+ datasets).
- Improved consistency/quality of reports.
- Improved patient management and outcomes.
- International audits of pathology practice based on an ICCR dataset.
- Adoption of ICCR pathology datasets by national oncology teams.
- Genuinely comparative data on cancer incidence and outcomes.

### What is the ICCR dataset process?

The established ICCR process for dataset development includes establishing an international expert panel of breast pathologists, with a surgeon and an oncologist on board. This team is tasked with drafting a set of core and noncore data items based on a critical review and discussion of current evidence. Commentary is provided for each data item to explain the rationale for selecting it as a core or noncore element, its clinical relevance, and to highlight potential areas of disagreement or lack of evidence, in which case a consensus position was formulated. Following international public consultation, the document is finalised, ratified, and the dataset, which includes a synoptic reporting guide, is published on the ICCR website.

The goal of the ICCR is to develop a set of data elements that will form the body of any pathology report on the specific cancer and which remain relevant and assessable in resource‐rich and resource‐poor countries. The datasets are layered to allow a combination of morphological and molecular items. Pathologists may add other elements when implementing or reporting. The intention is not to restrict them from adding items they feel are important to fit in with local practice.

### What are the key elements of an ICCR dataset?

Core elements are deemed essential for staging, clinical management, or prognosis of the cancer. These elements will either have high‐level evidentiary support (Level III) based on prognostic factors in the National Health and Medical Research Council (NHMRC) levels of evidence, 2 or above 1.^8^ In those circumstances, where level III 2 evidence is not available, an element may be core when there is unanimous agreement in the expert committee. An appropriate staging system, e.g. Pathological TNM staging, is normally included as a required element. The summation of all core elements is considered to be the reporting standard for a specific cancer.

Noncore elements have been unanimously agreed upon and should be included in the dataset but are not supported by level III 2 evidence. These elements may be clinically important and recommended as good practice, but are not yet validated or regularly used in patient management. They include information other than that which is essential for staging, clinical management, or prognosis, which are fundamental to the histological diagnosis and conclusion; for example, macroscopic observations and interpretation, block identification key, and others.

The commentary on data items is an explanatory text, diagrams, or tables that clarify the elements used. This defines the way an item should be reported, to ensure clarity and conformity, explaining why an item is included (e.g. how does the item assist with clinical management or prognosis of the specific cancer), cites published evidence in support of the element, and states any exceptions or issues. The commentary provides contextual guidance to the reporting pathologist.

### The ICCR invasive breast cancer datasets

There are four ICCR breast cancer related datasets, invasive carcinoma of the breast, with or without DCIS,^9^ DCIS and variants of lobular carcinoma *in situ* (LCIS) without invasive carcinoma, and microinvasive carcinoma (≤1 mm),^10^ surgically removed lymph nodes,^11^ and invasive breast cancer in the postneoadjuvant setting.^12^

## Highlights of the invasive breast cancer dataset with a focus on histological grade, tumour size measurement, and oestrogen receptor status

This review focusses on the invasive breast cancer dataset. The core and noncore elements are outlined in Table 1 and the commentary for each variable is available from the ICCR website and an overview publication (manuscript under review). Categorisation of histological type of invasive breast cancer aligns with the current WHO classification.^1^ Staging aligns with the AJCC UICC TNM system.^2^, ^3^ The commentary sections are very useful by clarification of the current evidence base, addressing any issues, and offering advice on how to assess each variable. The three variables with the largest commentary sections are (i) histological grade, (ii) tumour size measurement, and (iii) oestrogen receptor assessment.

**Table 1:** Core and noncore elements for the pathology reporting of invasive carcinoma of the breast based on the ICCR recommendations

Clinical information	Core (screen versus symptomatic presentation, nature of lesion, prior systemic treatment, imaging, family history)
Operative procedure	Core (wide local excision, mastectomy, lumpectomy, etc)
Specimen laterality	Core (right or left)
Tumour site	Core (quadrant or clockface)
Tumour focality	Core
	Noncore (number and size of each foci)
Tumour dimensions	Core (maximum dimension)
	Noncore (other dimensions)
Histological type	Core (according to the WHO classification)
Histological grade	Core (overall grade and its components)
Carcinoma *in situ*	Core (type, grade, ductal, lobular, encapsulated papillary carcinoma, solid papillary carcinoma *in situ*)
	Noncore (EIC)
Tumour extension	Core (skin, nipple, skeletal muscle)
Margin status	Core (invasive and DCIS involved, + distance to closest)
	Noncore (WLE ‐ distance to all margins)
LVI	Core
	Noncore (site if elsewhere)
Coexistent pathology	Noncore
Microcalcifications	Noncore
Oestrogen receptor	Core (+ve/−ve based on 1%, % or % range, average intensity)
Progesterone receptor	Core (+ve/−ve based on 1%, % or % range, average intensity)
HER2	Core (IHC score, ISH =/− ve, no cells counted, HER c/n, CEP17 c/n or Av no HER)
	Noncore (IHC % 3+ cells, ISH aneusomy, heterogeneity)
Ancillary studies	Noncore (eg Ki67, representative block for ancillary studies))
Pathological staging	Core (TNM)

Of the many core variables, histological grade has the most extensive commentary section, providing detailed international consensus recommendations on best practice, addressing issues where differences in practice and interpretation such as how to assess mitotic count, tubule, or gland formation have become apparent.

### Histological grade

The Nottingham histological grading (NHG) methodology^13^ for invasive breast carcinoma benefits from its simplicity and the key fact that it can be applied successfully to all forms of invasive breast cancer. It relies on human eye observation and translation of three core histological variables into numerical scores, which when summated provide a holistic overview of the biological characteristics and differentiation of a breast cancer. Its simplicity, however, is counterbalanced by the diversity of types of invasive breast carcinoma and their morphological heterogeneity, which can make application of NHG challenging in some circumstances and can lead to lack of concordance between pathologists. The ICCR dataset commentary provides the most detailed advice currently available to pathologists on how to determine NHG in their routine practice. This commentary benefits from being agreed upon and refined by a large international group of specialist breast pathologists followed by international peer review.

The method involves the assessment of three components of tumour morphology: tubule/acinus/gland formation, nuclear atypia/pleomorphism, and frequency of mitoses. Each is scored from 1 to 3. Adding the scores gives the overall histological grade, as shown below. The use of terms such as well differentiated or poorly differentiated in the absence of a numerical grade is considered inappropriate.

#### Assignment of glandular (acinar)/tubular differentiation score

Key points:

- In the assessment of gland/tubule formation, only structures in which there are clearly defined central lumens, surrounded by polarised tumour cells, should be counted. This does, however, include larger islands of tumour with central gland formation, as may be seen in mucinous carcinoma or invasive micropapillary tumours. Thus mucinous, micropapillary, and pure papillary tumours without, or with <10%, secondary luminal spaces are classified as having no tubular or glandular formation and assigned a score of 3.
- Papillary structures are not regarded as glandular/tubular structures.
- Intracytoplasmic lumen formation (intracytoplasmic vacuoles with true luminal microvillar surface, PAS+) does not count as gland formation, whatever the size of the intracytoplasmic vacuoles.

#### Assignment of nuclear pleomorphism score

Key points:

- Individual pathologists differ markedly in their approach to nuclear grading.^14^, ^15^
- Few cancers possess the very bland nuclei warranting an atypia/pleomorphism score of 1, and obvious atypia/pleomorphism should attract a score of 3.
- The minimum proportion of tumour nuclei that should show marked nuclear atypia/pleomorphism before a score of 3 is allocated has not been defined, but the finding of an occasional enlarged or bizarre nucleus should not be used to give a score of 3 rather than a score of 2.
- Features that can be used to assess atypia/pleomorphism include nuclear size, outlines, variation in the size and shapes, degree of nuclear chromasia, and nucleoli.
- Comparison with normal epithelial cell nuclei lining normal acini or ducts can help as control for nuclear size. If these are not present, red blood cells or lymphocytes can help to assess nuclear size.

#### Assignment of mitotic frequency score

Key points:

- Accurate mitosis counting requires high‐quality fixation, obtained when fresh specimens are sliced promptly after surgery and fixed immediately in neutral buffered formalin. This can be achieved without compromising the evaluation of resection margins.
- Poor quality fixation can result in underscoring of mitotic frequency.
- A minimum of 10 high power fields (HPFs) should be counted at the periphery of the tumour, where it has been demonstrated that mitotic activity is greatest on lower‐power search.
- If there is variation in the number of mitoses in different areas of the tumour, the least differentiated area (i.e. with the highest mitotic count) should be assessed.
- If the mitotic frequency score falls very close to a score cut point, one or more further groups of 10 HPFs should be assessed to establish the correct (highest) score.
- Fields chosen for scoring are selected during a random meander along the peripheral margin of the selected tumour area.
- Only fields with a representative tumour burden should be used. The low‐power scan of the tumour can be used to provide an assessment of the typical tumour‐to‐stromal ratio.
- Only definite mitotic figures (in any phase of the growth cycle) should be counted.^16^ Hyperchromatic nuclei and/or apoptotic nuclei should not be scored.
- The mitosis score depends on the number of mitoses per 10 HPFs. The size of HPFs of modern microscopes is very variable, so it is necessary to standardise the mitotic count. The scoring category should be assigned from the corresponding line of Table 2.
- Mitotic counts can also be expressed per mm^2^, which may be amenable to digital microscopy assessment. Modern microscopes have an HPF area which with 10 fields would equate to assessment of an area of ~2 mm^2^. Based on the current grading methodology, the cut points for number of mitoses identified in a tumour area of 2 mm^2^ is:
  - Mitotic score 1: ≤7
  - Mitotic score 2: 8–14
  - Mitotic score 3: ≥15

**Table 2:** Score categories according to field diameter, area, and mitotic count

Field diameter (mm)	Area (mm^2^)	Number of mitoses per 10 fields corresponding to:		
		Score 1	Score 2	Score 3
Scoring categories of mitotic counts				
0.40	1.25	≤4	5 to 9	≥10
0.41	0.132	≤4	5 to 9	≥10
0.42	0.139	≤5	6 to 10	≥11
0.43	0.145	≤5	6 to 10	≥11
0.44	0.152	≤5	6 to 11	≥12
0.45	0.159	≤5	6 to 11	≥12
0.46	0.166	≤6	7 to 12	≥13
0.47	0.173	≤6	7 to 12	≥13
0.48	0.181	≤6	7 to 13	≥14
0.49	0.189	≤6	7 to 13	≥14
0.50	0.196	≤7	8 to 14	≥15
0.51	0.204	≤7	8 to 14	≥15
0.52	0.212	≤7	8 to 15	≥16
0.53	0.221	≤8	9 to 16	≥17
0.54	0.229	≤8	9 to 16	≥17
0.55	0.238	≤8	9 to 17	≥18
0.56	0.246	≤8	9 to 17	≥18
0.57	0.255	≤9	10 to 18	≥19
0.58	0.264	≤9	10 to 19	≥20
0.59	0.273	≤9	10 to 19	≥20
0.60	0.283	≤10	11 to 20	≥21
0.61	0.292	≤10	11 to 21	≥22
0.62	0.302	≤11	12 to 22	≥23
0.63	0.312	≤11	12 to 22	≥23
0.64	0.322	≤11	12 to 23	≥24
0.65	0.332	≤12	13 to 24	≥25
0.66	0.342	≤12	13 to 24	≥25
0.67	0.353	≤12	13 to 25	≥26
0.68	0.363	≤13	14 to 26	≥27
0.69	0.374	≤13	14 o 27	≥28

Reproduced with permission from The Royal College of Pathologists (2016). *Pathology reporting of breast disease in surgical excision specimens incorporating the dataset for histological reporting of breast cancer*. The Royal College of Pathologists and National Coordinating Committee for Breast Pathology.

#### Important issues to be aware of when assessing histological grade

**Audit**: Published ratios for grades 1, 2, and 3 are approximately 2:3:5 in symptomatic breast cancer, with about half of all symptomatic cancers assigned as grade 3.^17^ Screen‐detected cancer series are likely to include a smaller proportion of high‐grade cases.

**Fixation**: Poor fixation impairs accurate assessment of mitotic frequency, reducing their visibility, which can result in a change in grade ratios, typically with a larger proportion of grade 2 cases and a lower proportion of grade 3 cases. If audit of grade distribution in symptomatic cancers shows substantially fewer grade 3 cases, or a majority of grade 2 cases, fixation and grading protocols should be reviewed.

**Heterogeneity**: Some degree of variation in appearance from one part of a tumour to another undoubtedly occurs; this is particularly true of tumours of mixed type. Nuclear appearances and mitotic figures are evaluated at the periphery and/or least differentiated and solid areas of the tumour.

### Tumour size measurement

The size of the single or of the largest/dominant invasive tumour focus is a key variable required for breast cancer staging and requires accurate assessment to the nearest mm. Histological tumour size is deemed the gold standard but should be correlated with the gross macroscopic size measurement and where possible with the imaging size.

Key points:

- In the context of extensive surrounding DCIS (and/or) florid or pleomorphic LCIS, the total extent of the entire disease process including all invasive tumour foci and associated DCIS should be provided as the whole tumour size. This information is useful for clinical and radiological correlation and to assist in the determination of completeness of disease excision.
- In the context of multiple invasive tumours without associated extensive DCIS, the total extent of disease can be used to indicate the total size of area involved by invasive carcinoma.
- For more complex tumours, such as synchronous primary carcinomas in separate quadrants, a pragmatic description of each tumour and its accompanying features will be necessary.
- Where microscopic size measurement is not possible or deemed inaccurate, for example prior needle biopsy partial removal or piecemeal resection of the tumour at single or multiple operations, the gross macroscopic, imaging and clinical tumour size, listed here in priority sequential order, should be used.
- It is recognised that the distinction between a separate satellite invasive tumour focus based on a distance of 5 mm or greater is arbitrary, but this distance has been accepted as a pragmatic approach.

### Hormone receptor status

Hormone receptor status is determined primarily to identify patients who may benefit from endocrine therapy. Currently, oestrogen receptor (ER) status is used to select patients suitable for endocrine therapy. Progesterone receptor (PR) status has been shown to have prognostic value and to provide information on the degree of response to endocrine therapy in patients with ER‐positive tumours. About 75–80% of invasive breast cancers are positive for ER and PR.

Key points:

- Hormone receptor status is most often determined in formalin‐fixed, paraffin‐embedded tissue sections by immunohistochemistry (IHC). Only nuclear staining is considered positive. Single‐gene expression assays are not recommended for routine use.
- Definition of a positive result: Studies using both IHC and the ligand binding assay suggest that patients with higher hormone receptor levels have a higher probability of response to endocrine therapy, but expression as low as 1% positive staining has been associated with a clinical response. As a result, the guidelines recommend classifying all cases with at least 1% positive cells as receptor‐positive. All current guidelines recommend that carcinomas with <1% positive cells be considered negative for ER and PR.
- ER low positive: It has become increasingly recognised that there are limited data on response to endocrine therapy in carcinomas with low‐level ER expression, defined as 1–10% positive cells, although the available information currently supports possible benefit. It is recommended that these tumours remain classified as positive and considered eligible for endocrine treatment, but be designated Low ER Positive. For patients with low ER expression the decision on endocrine therapy should be based on an analysis of its risks and potential benefits.
- Use of hormone receptor scoring systems such as Allred, Quick score, and H score are optional.

## Conclusion

Structured pathology reporting in the form of a synoptic report ensures that data are complete, promotes easy access and communication to clinical teams and, when coded, can facilitate automated transfer to cancer registries. The ICCR dataset is the first internationally agreed dataset for the reporting of resection specimens with invasive cancer of the breast and which aims to improve the quality of invasive breast cancer reporting globally, regardless of available resources. Worldwide standardised reporting, aligning with the WHO blue book tumour classification, will allow the establishment of benchmarking metrics that define good practice.

## Author contributions

**Ian Ellis**, drafted the article, reviewed, and approved the final version. Gary Tse reviewed, and approved the final version of the article. Puay Hoon Tan reviewed, and approved the final version of the article. Emad Rakha reviewed, and approved the final version of the article.

## Ethical approval and consent to participate

N/A.

## Funding

N/A.

## Conflict of interest

The authors have no conflicts of interest to declare.

## Data Availability

Data sharing is not applicable to this article, as no new data were created or analysed in this study.
